# The Link of Three-Dimensional Frailty Index With Quality of Life and Fear of Falling Among Taiwanese Older Adults

**DOI:** 10.1093/geroni/igaa057.966

**Published:** 2020-12-16

**Authors:** Duan-Rung Chen, Chun-Tung Kuo, Peng-Yu Chen

**Affiliations:** 1 National Taiwan University, Taipei, Taiwan (Republic of China); 2 institute of Health Behaviors and Community Sciences, Taipei, Taiwan (Republic of China)

## Abstract

Objective. Frailty has received increasing attention as a way of understanding gradual losses in one or more domains of human functioning (physical, psychological, and social) in older adults. Studies suggested that frailty is related to lower quality of life (QoL) and the fear of falling (FoF). The most commonly used frailty criteria is the Fried Phenotype, which solely focuses on physical dimension of frailty. This study aims to evaluate the three-dimensional frailty index (namely, physical, psychological and social), and its association with QoL and FoF in a sample of community-dwelling Taiwanese older people. Methods. A total of 751 older adults aged 65 years and older (mean age 73.69 yrs ; SD=6.6) were included from May 2019 to Jan 2020 in Taipei City. The 8-Item Short-Form Health and the Falls Efficacy Scare International (FES-I) were used. Structural equation models (SEM) were employed to examine the association of the three-dimensional frailty index with QoL and FoF. Results. The SEM results confirmed a three-dimensional frailty index (physical, psychological and social frailty), and it is significantly associated with OoL and FoF. Physical frailty had the strongest association with PCS and FES-I, yet social frailty with MCS. Conclusion. Public health efforts to prevent elderly frailty should not solely focusing on physical aspect of frailty.

